# *Wld^S ^*but not Nmnat1 protects dopaminergic neurites from MPP^+ ^neurotoxicity

**DOI:** 10.1186/1750-1326-7-5

**Published:** 2012-02-08

**Authors:** Jo Ann V Antenor-Dorsey, Karen L O'Malley

**Affiliations:** 1 Department of Anatomy and Neurobiology, Washington University School of Medicine, Saint Louis, MO, 63110, USA

**Keywords:** *Wld^S^*, Nmnat1, Parkinson's disease, MPP^+^, dopaminergic neurons, axonal degeneration

## Abstract

**Background:**

The *Wld^S ^*mouse mutant ("Wallerian degeneration-slow") delays axonal degeneration in a variety of disorders including *in vivo *models of Parkinson's disease. The mechanisms underlying *Wld^S ^*-mediated axonal protection are unclear, although many studies have attributed *Wld^S ^*neuroprotection to the NAD^+^-synthesizing Nmnat1 portion of the fusion protein. Here, we used dissociated dopaminergic cultures to test the hypothesis that catalytically active Nmnat1 protects dopaminergic neurons from toxin-mediated axonal injury.

**Results:**

Using mutant mice and lentiviral transduction of dopaminergic neurons, the present findings demonstrate that *Wld^S ^*but not Nmnat1, Nmnat3, or cytoplasmically-targeted Nmnat1 protects dopamine axons from the parkinsonian mimetic N-methyl-4-phenylpyridinium (MPP^+^). Moreover, NAD^+ ^synthesis is not required since enzymatically-inactive *Wld^S ^*still protects. In addition, NAD^+ ^by itself is axonally protective and together with *Wld^S ^*is additive in the MPP^+ ^model.

**Conclusions:**

Our data suggest that NAD^+ ^and *Wld^S ^*act through separate and possibly parallel mechanisms to protect dopamine axons. As MPP^+ ^is thought to impair mitochondrial function, these results suggest that *Wld^S ^*might be involved in preserving mitochondrial health or maintaining cellular metabolism.

## Background

Parkinson's disease (PD) is the second most common neurodegenerative disorder in the U.S., affecting 1-2% of people over the age of 55. Characterized by loss of dopaminergic neurons in the substantia nigra (SN) [1,2], the cardinal motor symptoms of PD include resting tremor, bradykinesia, rigidity, and abnormal gait [3,4]. Another characteristic of PD is its late onset and progressive nature. Symptoms appear after 50-70% [5,6] of striatal dopamine has been depleted and 30-50% [7,8] of the nigral dopaminergic cells have died. Such studies suggest that the extent of striatal dopamine depletion is better correlated with the severity of PD symptoms than the loss of dopaminergic neurons in the SN [7].

Data from PD-linked genetic mutations also support the notion that axonal pathology and/or dysfunction occurs prior to the loss of dopaminergic cell bodies. For example, α-synuclein pathology is seen in neurites before it is observed in PD-associated cell bodies [3,9]. α-synuclein mutants accumulate in the cell soma when overexpressed in cortical neurons, suggesting impaired axonal transport as well [10]. Moreover, transgenic models expressing the PD-linked mutant gene leucine rich repeat kinase 2 (LRRK2) also exhibit pronounced axonal loss and pathology prior to cell body loss [11]. In addition, genetic mutations in other PD-linked genes such as Parkin, an E3 ligase [12], and PINK1 (PTEN-induced putative kinase 1 protein) a mitochondrially-targeted kinase, also alter axonal transport [13,14]. Collectively, these findings have led to the idea that nigral neurons degenerate through a "dying back" axonopathy where degeneration starts in the distal axon and proceeds over time towards the cell body.

Environmental toxins known to mimic PD such as rotenone and MPP^+ ^also disrupt axonal function. These factors not only inhibit mitochondrial Complex I activity, but also de-polymerize microtubules leading to axon fragmentation and decreased synaptic function [15-17]. Moreover, MPP^+ ^can directly inhibit axon transport in the squid axoplasm [18] and DA neurons [19]. Thus, results from PD-associated environmental and genetic factors support an early, critical role for axonal impairment in PD.

Recent data suggest that the Wallerian degeneration slow fusion protein (*Wld^S^*) can delay axonal degeneration about 10-fold from a wide variety of genetic and toxin-inducing stimuli in the peripheral nervous system [20]. *Wld^S ^*also blocks axon degeneration in several central nervous system (CNS) models of degeneration including animal models of PD [21,22]. For example, we previously found that *Wld^S ^*rescues 85% of dopaminergic axons for at least 7 days post MPTP treatment *in vivo *[23]. Because no other mutation or drug protects axons as robustly as *Wld^S^*, understanding how the *Wld^S ^*fusion protein is able to prevent axon degeneration is the first step towards defining an intervention that would leave axons intact.

*Wld^S ^*is a chimeric protein composed of the first 70 amino acids of the ubiquitination factor E4b (Ube4b) followed by an 18-amino acid linker region and then the entire coding sequence for nicotinamide mononucleotide adenylyltransferase (Nmnat1), a nicotinamide adenine dinucleotide (NAD^+^) synthesizing enzyme [24,25]. Most studies suggest that catalytically active Nmnat1 is necessary for axonal protection [26,27], hence, exogenous addition of NAD^+ ^has been reported to delay Wallerian degeneration in response to axotomy in dorsal root ganglion (DRG) cells [28]. In *Drosophila*, however, the picture is more complex in that Avery *et al. *[29], showed that Nmnat enzymatic activity is required following axotomy whereas Zhai *et al. *[30] found that Nmnat does not need its catalytic domain to protect axons. In this model [30], as well as in a new study demonstrating that Nmnat also protects dendrites [31], Nmnat exhibits a separate chaperone-like activity which protects axons and dendrites [30,32].

Inasmuch as most studies have been done in peripheral model systems and because we have previously shown that *Wld^S ^*protects dopaminergic terminal fields from MPTP *in vivo*, we used a dissociated midbrain culture system to determine the mechanism of *Wld^S^*-mediated neurite protection in dopamine neurons. Here, we show that, regardless of its enzymatic activity, the entire *Wld^S ^*sequence is needed for the *Wld^S^' *neuroprotective phenotype in dopaminergic neurons. Our data also illustrate that NAD^+ ^has a neuroprotective effect that is different from *Wld^S^*-mediated protection.

## Results

### *Wld*^*S *^protects cell bodies and neurites from MPP^+^

**Figure 1:**
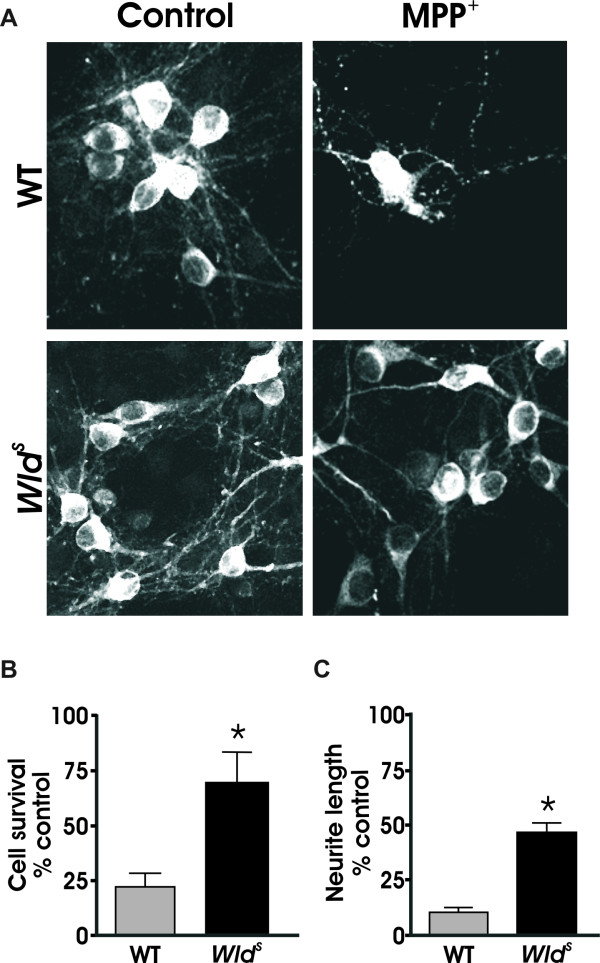
***Wld^s ^*protects dopaminergic neurons from MPP^+ ^*toxicity***. (A) Dissociated dopaminergic cultures from both WT and *Wld^s ^*mice were treated with 2 μm MPP^+ ^for 48 hours, and processed for TH immunoreactivity. (B) Quantification of TH+ cell bodies and (C) TH+ neurites was done using unbiased stereology. Data are normalized to control cultures and denote the mean ± SEM of representative determinations made in three separate cultures. *p < 0.01; **p < 0.001.

Previously we have shown that dopaminergic terminal fields but not cell bodies of *Wld^S ^*mice are protected against MPTP injury [23]. To confirm and extend these observations in a more tractable system, we utilized dissociated cultures of midbrain neurons in which 1-5% of the total cells plated are dopaminergic [33]. Results show that cultures from *Wld^S ^*mice exhibited significant protection of neurites not seen in wild type cultures after MPP^+ ^treatment (Figure 1A, C). Moreover, dopaminergic cell death from MPP^+ ^treatment was also attenuated in *Wld^S ^*cultures, unlike those seen *in vivo *(Figure 1A, B). Thus *Wld^S ^*can effectively protect neurites (dendrites and axons) as well as cell bodies from known PD mimetics *in vitro*.

### Cytoplasmic *Wld*^*S *^protects cell bodies and neurites against MPP^+^

**Figure 2:**
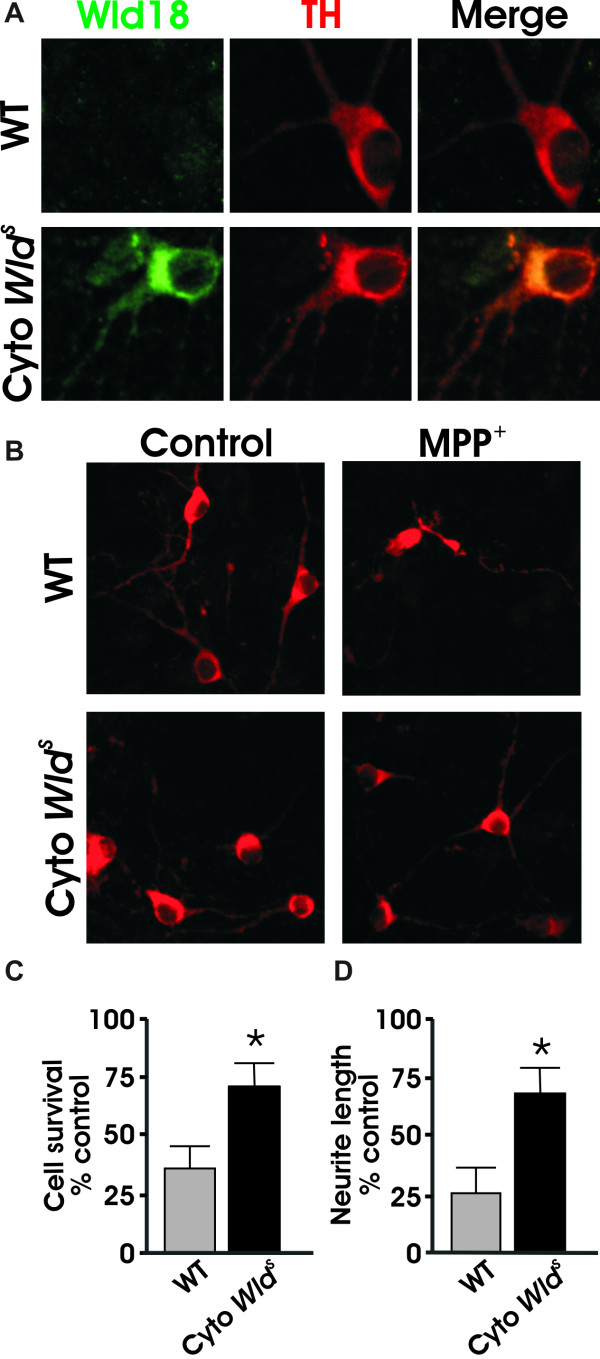
**Cytoplasmic *Wld^s ^*protects dopaminergic neurons from MPP^+ ^toxicity**. (A) Dissociated dopaminergic cultures from both WT and cyto *Wld^s ^*mice were co-stained with TH and *Wld^s ^*antibodies to confirm the subcellular localization of *Wld^s^*. (B) Cultures were treated with 2 μm MPP^+ ^for 48 hours prior to fixing and staining. (C) Quantification of TH+ cell bodies and (D) TH+ neurites shows that cytoplasmic *Wld^S ^*protected both cell bodies and neurites against MPP^+^. Data are normalized to control cultures and denote the mean ± SEM of representative determinations made in three separate cultures. *p < 0.05.

Recent studies have reported that the localization of *Wld^S ^*influences its neuroprotective effect. Babetto *et. al*. have reported that a cytoplasmic version of the *Wld^S ^*protein (cyto *Wld^S^*) confers a higher level of protection than the native form of *Wld^S ^*[34]. In addition, Sasaki *et. al*. have reported that cytoplasmic Nmnat1 (cyto Nmnat1) and Nmnat3, which is primarily localized in the mitochondria, also confer a higher level of protection than Nmnat1 [35,36]. To test whether cytoplasmic localization of *Wld^S ^*rescued or changed the level of protection seen with nuclear *Wld^S^*, primary dopaminergic neurons were prepared from wild type and cyto *Wld^S ^*mice, treated with MPP^+^, and analyzed as described. Results show that cyto *Wld^S ^*mice exhibited a similar level of protection of dopaminergic cell bodies and neurites as seen in *Wld^S ^*mice (Figure 2). Therefore, as reported for peripheral model systems and certain CNS paradigms [37], redirecting most of Wld^S ^from the nucleus to the cytoplasm protects processes equally well.

### Nmnat1 does not protect against MPP^+ ^toxicity

**Figure 3:**
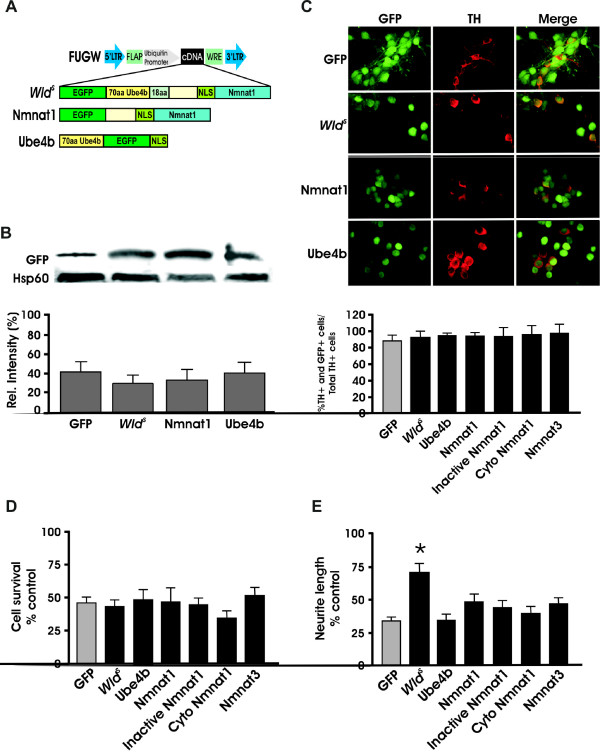
**Nmnat by itself does not protect dopaminergic neurons from MPP^+ ^toxicity**. (A) Diagram of the lentiviral constructs used to transduce WT dissociated dopaminergic neurons. (B) Western blot of cell lysates from transduced primary midbrain cultures illustrates that all the transduced transgenes exhibit similar levels of expression. (C) Similar transduction efficiencies of the different lentiviruses were confirmed by quantifying the number of TH+ and GFP+ cells following transduction of dopaminergic cultures. (D) Quantification of TH+ cell bodies and (E) TH+ neurites show that only *Wld^S^*-transduced cultures protected neurites against MPP^+^. Data are normalized to control cultures and denote the mean ± SEM of representative determinations made in three separate cultures. *p < 0.001.

Many studies, especially in peripheral model systems, have shown that Nmnat1 can at least partially mimic the effects of *Wld^S ^*[26,27]. To determine whether this is true in dopaminergic neurons, we transduced primary midbrain cultures from wild type animals with either GFP, Nmnat1, the 70 amino acid fragment of Ube4b encoded within the *Wld^S ^*gene, or the entire *Wld^S ^*coding region using lentiviral vectors expressing GFP [27] (Figure 3). We also tested the effects of Nmnat3, cytoplasmic Nmnat1, and enzymatically inactive Nmnat1 (Nmnat1 (W170A)) [28,38] (Figure 3D, E and Additional File 1 Figure S1C, D). Immunofluorescence and western blotting was done to confirm that transductions led to similar expression levels in dissociated cultures (Figure 3B, C, Additional File 1 Figure S1B, D-E). Despite equivalent levels of transgene expression, only neurites transduced with the entire coding sequence of *Wld^S ^*were protected from MPP^+ ^injury (Figure 3D, E).

**Figure 4:**
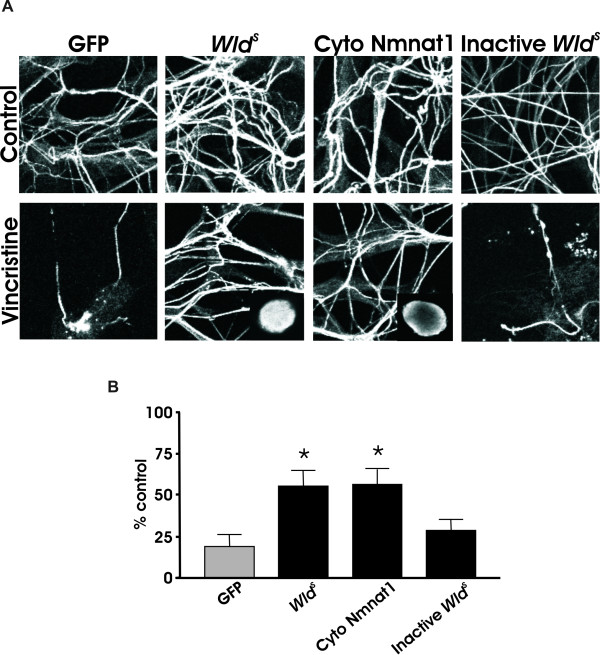
***Wld^s ^*and cytoplasmic Nmnat1 protect DRG axons from vincristine toxicity**. (A) DRG cultures from E14 mice transduced with GFP, *Wld^s^*, cyto Nmnat1, or inactive *Wld^s ^*were processed for acetylated tubulin immunoreactivity 24 hours after vincristine treatment. Inserts in bottom middle panels show 40× images of DRG cell bodies transduced with *Wld^s ^*and cytoplasmic Nmnat1, respectively, to illustrate no overt nuclear enrichment of Nmnat1. (B) Quantification of neurites shows that both *Wld^s ^*and cyto Nmnat1 protects DRG neurites from vincristine toxicity. Data are normalized to control cultures and denote the mean ± SEM of representative determinations made in three separate cultures. *p < 0.05.

Because many studies have suggested that Nmnat and in particular cyto Nmnat or axonally targeted Nmnat can be as effective as *Wld^S ^*in protecting axons from mechanical or toxic insults, we used DRG cultures as a positive control [34,39]. Consistent with those studies, both *Wld^S ^*and cyto Nmnat rescued DRG neurites from the neurotoxin, vincristine, whereas the GFP-only and inactive *Wld^S ^*virus did not (Figure 4). Taken together, these data confirm previous results showing that cyto Nmnat is necessary and sufficient to save DRG neurites. In contrast, only *Wld^S ^*but not cyto Nmnat, Nmnat1, or Nmnat3 was able to protect dopaminergic neurons from the neurotoxic effects of MPP^+^.

### Inactive *Wld*^*S *^protects cell bodies and neurites against MPP^+^

**Figure 5:**
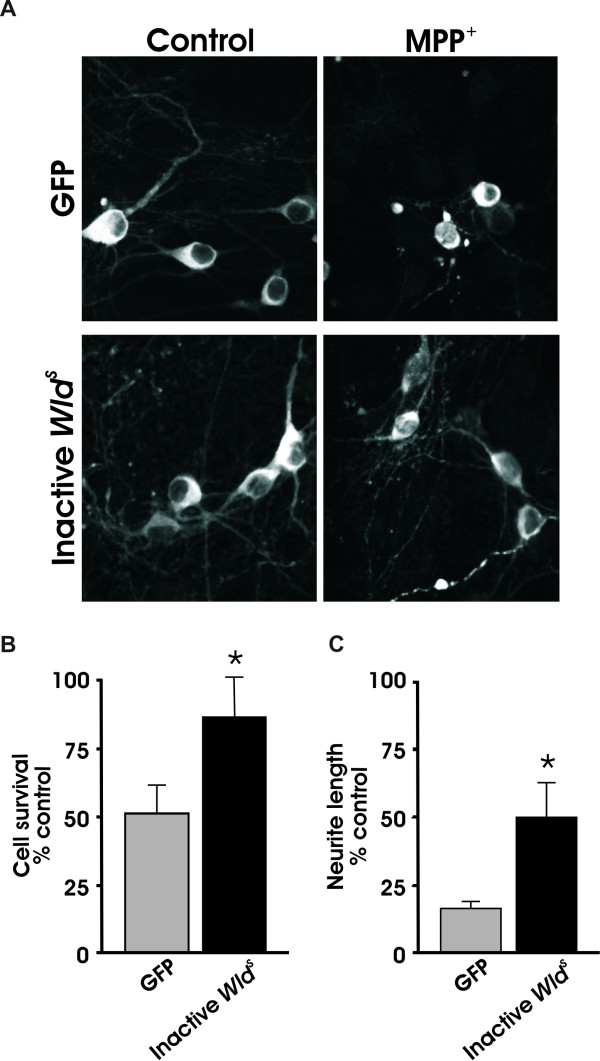
**Inactive *Wld^s ^*also protects dopaminergic neurons from MPP^+ ^toxicity**. (A) Dissociated dopaminergic neurons transduced with GFP or inactive *Wld^s ^*were treated and processed as described. (B) Quantification of TH+ cell bodies and (C) TH+ neurites. Data are normalized to control cultures and denote the mean ± SEM of representative determinations made in three separate cultures. *p < 0.05.

To corroborate the hypothesis that Nmnat1 does not protect dopaminergic neurons from MPP^+^, we transduced dissociated primary midbrain cultures with enzymatically inactive *Wld^S ^*(W258A; [27]). In contrast to our own results in DRG cultures (Figure 4) as well as results published by others using this same construct [27], the inactive *Wld^S ^*plasmid was as effective as NAD^+^-synthesizing *Wld^S ^*animals in protecting dopaminergic cell bodies and neurites against MPP^+ ^injury (Figure 5). Therefore, the entire *Wld^S ^*chimeric protein, but not its NAD^+^-synthesizing activity, is required for neuroprotection of dopaminergic neurons.

### *NAD*^+ ^protects cell bodies and neurites against MPP^+^

**Figure 6:**
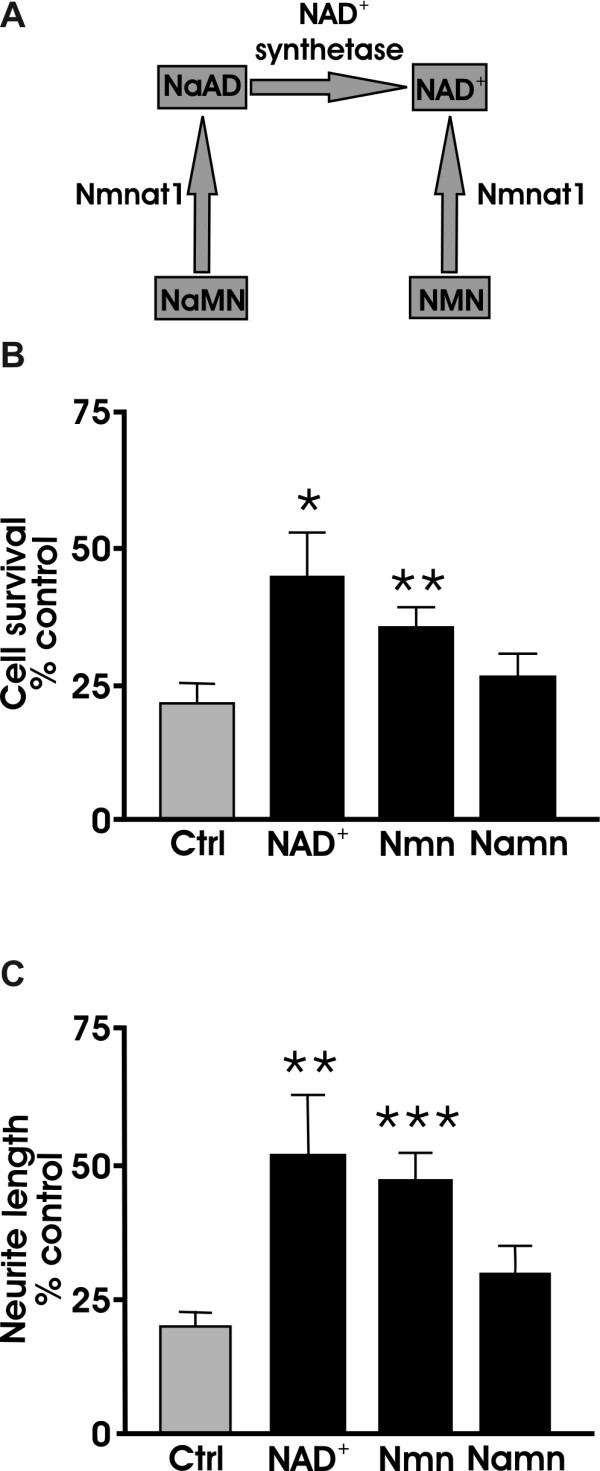
**NAD^+ ^protects dopaminergic cells and neurites from MPP^+ ^toxicity**. (A) NAD^+ ^biosynthetic pathway [75]. (B) Dissociated WT dopaminergic cultures were pretreated with NAD^+^, Nmn, or Namn 24 hours before addition of 2 μm MPP^+^. Quantification of TH+ cell bodies and (C) TH+ neurites show that NAD^+ ^and Nmn, but not Namn, protected cells and neurites from MPP^+^. Data are normalized to control cultures and denote the mean ± SEM of representative determinations made in three separate cultures. *p < 0.05, **p,0.01, ***p < 0.001.

Previous studies have shown that NAD^+ ^itself can be neuroprotective [27]. Although Nmnat1 by itself did not recapitulate the neuroprotective effect of *Wld^S ^*on dopaminergic neurons, we tested whether NAD^+ ^or one of its precursors (Figure 6A) rescued cell bodies or neurites from MPP^+ ^treatment. Therefore, dissociated dopaminergic wild type cultures were pretreated with either 1 mM of NAD^+^, nicotinamide mononucleotide (NMN), or nicotinic acid mononucleotide (NaMN) 24 hours before MPP^+ ^treatment. Both NAD^+ ^and NMN but not NaMN protected cell bodies and neurites against MPP^+ ^toxicity (Figure 6B, C). These findings together with the results showing that catalytically-inactive *Wld^S ^*was able to protect dopamine neurons (Figure 5) but catalytically active Nmnat did not (Figure 3F) suggest that different pathways are being invoked in response to MPP^+ ^toxicity.

### Sirt1 is not responsible for the NAD-mediated protection of cell bodies and neurites against *MPP*^+^

**Figure 7:**
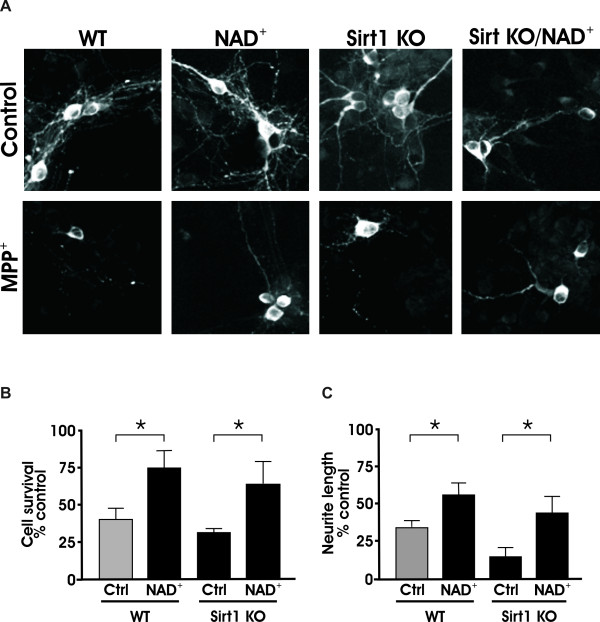
**NAD^+ ^does not protect dopaminergic neurons through the Sirt1 pathway**. (A) Dissociated midbrain cultures from both WT and Sirt1 KO mice were pretreated with NAD^+ ^24 hours before addition of 2 μm MPP^+^. (B) Quantification of TH+ cell bodies and (C) TH+ neurites show that NAD^+ ^protects cells and neurites from MPP^+ ^in both WT and Sirt1 KO cultures. Data are normalized to control cultures and denote the mean ± SEM of representative determinations made in three separate cultures. *p < 0.05.

Previous studies in DRG neurons have attributed the protective phenotype of *Wld^S ^*to its action on the Nmnat1-NAD^+^-Sirt1 pathway [27]. To test the involvement of Sirt1, we prepared dissociated dopaminergic cultures from Sirt1 knockout mice. Following 24 hour pretreatment with 1 mM NAD^+ ^or vehicle control, cultures were exposed to MPP^+^. Consistent with the notion that NAD^+ ^is not acting through Sirt1 but rather through a different mechanism, NAD^+ ^protected cell bodies and neurites in Sirt1 knockout cultures from MPP^+ ^toxicity (Figure 7).

### *NAD*^+ ^and *Wld*^*S *^effects are additive

**Figure 8:**
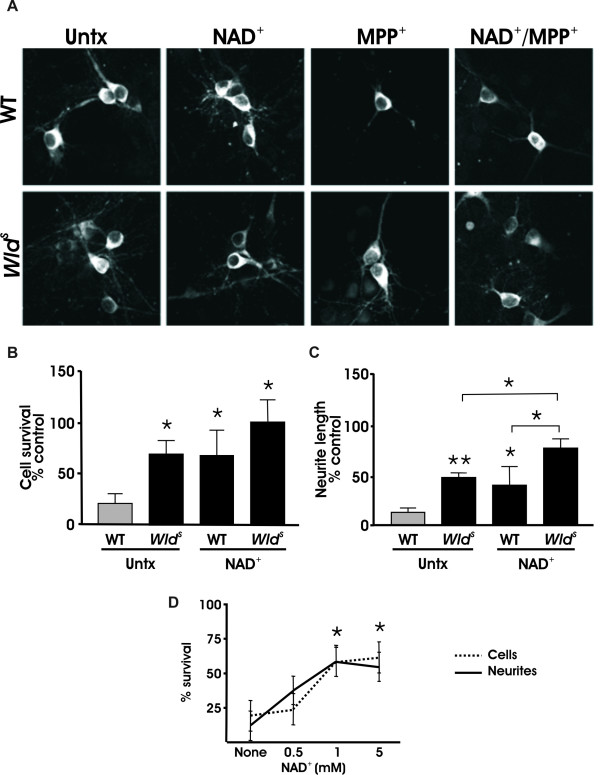
**The protective effect of NAD^+ ^and *Wld^S ^*are additive**. (A) Dissociated midbrain cultures from both WT and *Wld^S ^*mice were pretreated with NAD^+ ^24 hours before addition of 2 μm MPP^+^. (B) Quantification of TH+ cell bodies and (C) TH+ neurites show that NAD^+ ^pretreatment was more effective in protecting *Wld^S ^*neurites from MPP^+ ^versus untreated *Wld^S ^*cultures. (D) NAD^+ ^dose response curve showing that the protection seen with 1 mM NAD^+ ^is maximal. Addition of 10 mM NAD^+ ^before MPP^+ ^treatment induced 50% cell death in dopaminergic neurons (data not shown). Data are normalized to control cultures and denote the mean ± SEM of representative determinations made in three separate cultures. *p < 0.05; **p < 0.001.

The data described above suggest that *Wld^S ^*is acting through a separate possibly parallel pathway from that of NAD^+ ^in dopaminergic neurons. If so, then adding NAD^+ ^to *Wld^S ^*cultures will enhance the neuroprotective phenotype of *Wld^S^*. To see whether the NAD^+ ^effect overlapped with *Wld^S ^*or was additive, dissociated dopaminergic cultures were prepared from wild type and *Wld^S ^*mice and pre-treated with and without NAD^+ ^as previously described. Both NAD^+ ^and *Wld^S ^*alone exhibited similar levels of cell body and neurite protection (Figure 8). However, NAD^+ ^together with *Wld^S ^*generated significantly higher levels of protection suggesting this is an additive process (Figure 8). To test whether these effects were maximal, additional cultures were treated with 5 mM NAD^+^; no significant differences in neuroprotection were observed when compared with the lower dose of 1 mM NAD^+ ^(Figure 8D). These findings demonstrate that NAD^+ ^and *Wld^S ^*are additive in the MPP^+ ^model suggesting that they are acting through separate and possibly parallel neuroprotective mechanisms.

## Discussion

The mechanism(s) by which *Wld^S ^*protects axons is still unclear. Peripheral model studies underscore the role of Nmnat and its product, NAD^+^, in protecting axons from various injuries whereas few central nervous system studies have been done. Using cellular, molecular and pharmacological tools, the present findings show that the chimeric *Wld^S ^*gene product plays a critical role in protecting dopaminergic processes, one not dependent upon Nmnat activity. Specifically, neither Nmnat, cytoplasmically-targeted Nmnat, nor Nmnat 3 were able to prevent toxicity associated with the dopaminergic toxin MPP^+ ^whereas, akin to previous reports [27,28], cyto Nmnat protected DRG axons from known axonal toxins. In contrast, *Wld^S^*, cytoplasmically-expressed *Wld^S^*, and *Wld^S ^*with an inactive Nmnat domain, all significantly protected dopaminergic neurites from toxin-mediated loss. Despite the inability of Nmnat to protect dopamine processes, NAD^+ ^and its precursor, Nmn, were neuroprotective. As *Wld^S ^*and NAD^+ ^were additive in this model system, current results suggest that these protectants act through separate, possibly parallel pathways. This is in agreement with previous findings by Wishart *et. al*. (2008) [40] showing that *Wld^S ^*increases expression of cell cycle-related genes through both NAD^+^-dependent and independent pathways. Thus, NAD^+ ^or its derivatives as well as *Wld^S ^*and its targets protect dopamine processes and may aid in the development of therapeutics preserving the connections and circuitry important in PD.

The role of Nmnat and NAD^+ ^in recapitulating the full effect of *Wld^S ^*has been controversial. *In vitro *studies have shown that overexpression of Nmnat1 by itself protects axons from many mechanical, genetic or toxin-induced injuries [20,41]. In contrast, transgenic animals expressing nuclear Nmnat1 did not replicate the effects of *Wld^S ^*[42,43] whereas cytoplasmically [39] or axonally targeted Nmnat1 [34] were equally if not more effective. Thus, site of action plays a role in Nmnat1's effectiveness [20]. These data together with findings showing that the first 16 N-terminal amino acids of the *Wld^S ^*gene product are required for full *Wld^S ^*protection [26], possibly by redistributing enough *Wld^S ^*to cytoplasmic or axonal compartments, are consistent with the notion that both the N-terminal portion of *Wld^S ^*and Nmnat1 are necessary for full axonal protection [20].

The importance of Nmnat catalytic activity is reflected in several mutational studies in which Nmnat's active sites have been disrupted and neuroprotection was lost [26,27,29,43]. Moreover, NAD^+ ^itself and/or some of its biosynthetic precursors, protect against axonal degeneration in peripheral model systems as well as in experimental autoimmune encephalomyelitis (EAE); [28,44], ischemia [45,46], Alzheimer's disease [47], and PD [48-50]. In at least one study however, addition of NAD^+ ^was not effective [42]. Moreover, Drosophila Nmnat (dNmnat) did not require enzymatic activity for axon protection against insults such as excitotoxicity, polyglutamine-induced dysfunction, or mechanical injury [32] leading to the suggestion that dNmnat may perform a chaperone-like function [30]. Indeed, structural studies of various Nmnats have revealed characteristic similarities to known chaperones such as UspA and Hsp100 [51]. Consistent with this notion, dNmnat was recently shown to function as a stress protein in response to heat shock, hypoxia, and the mitochondrial Complex I toxin, paraquat [52]. However, in dopaminergic neurons, Nmnat1 does not seem to function as either an axonal protectant or a chaperone.

Studies have indicated that MPP^+ ^can block electron transport by acting at the same site as the Complex I inhibitor, rotenone, leading to the production of free radical species and a loss of ATP production [53-55]. MPP^+ ^affects other processes as well including the rapid release of dopamine from vesicular stores [56,57]; depolymerization of microtubules [16,58]; induction of autophagy [19,59], and the rapid loss of mitochondrial membrane potential and reduction in mitochondrial motility in dopamine axons [19]. Since many of these effects involve mitochondrial function, conceivably the *Wld^S ^*gene product is involved in preserving mitochondrial health or maintaining homeostatic control. Recently, Barrientos *et al. *reported that *Wld^S ^*is able to regulate the mitochondrial permeability transition pore (PTP) preventing, amongst other things, calcium release, ATP loss, oxidative stress and release of proteins involved in axonal degeneration [60]. This is consistent with Wishart *et al. *(2007) showing that synaptosomes isolated from *Wld^S ^*versus wild type animals expressed higher levels of various mitochondrial proteins including the PTP protein, VDAC2 [61]. Barrientos *et al. *suggested that *Wld^S ^*is part of a regulatory cascade that also involves JNK activation upstream of PTP opening [60]. Although JNK is a known regulator of axon degeneration in DRGs [62], it has been reported to not play a role in 6-OHDA-mediated degeneration of the striatum [7]. In addition, we have recently showed that the JNK inhibitor, SP600125, did not prevent MPP^+ ^effects on dopaminergic mitochondria [19]. Thus diverse, unknown, regulatory steps appear to mediate *Wld^S ^*effects in dopamine axons.

Given its role as a ubiquitous cofactor, NAD^+ ^influences many cellular decisions such as DNA damage repair [63] and transcriptional regulation and differentiation [64]. Earlier studies suggested that increased NAD^+ ^levels led to SIRT activation which, in turn, activated a transcription factor that induced genes involved in neuroprotection [27,32]. Although an attractive hypothesis, subsequent studies using SIRT1 knock out animals did not support this notion for DRG neurons [65], or as in the present study, for dopaminergic neurons (Figure 8).

Unlike what we have reported *in vivo *[23], dopaminergic cell death from MPP^+ ^treatment was also attenuated in *Wld^S ^*cultures (Figure 1). Given that *Wld^S ^*is known to protect axons and synapses from injury, it is possible that it can also indirectly protect cell bodies. Similar indirect effects on cell bodies have been reported by Gillingwater *et. al*. (2004) in both the caudate nucleus and hippocampus of *Wld^S ^*mice following transient global ischemia [66]. More recently, a cytoplasm-targeted Nmnat transgenic mouse protected cell bodies and processes from NMDA-mediated excitotoxicity [37]. Authors of the latter study speculate that Nmnat can potentially influence a common pathway, albeit one not tied to caspase 3 activation, in certain neurons. Perhaps a similar pathway is activated in other systems as well since *Wld^S ^*also protects motoneuron cell bodies in a mouse model of progressive motor neuropathy [67].

Why are results in dopamine neurons different than other systems? Because many of the studies published have been performed in peripheral model systems with dramatically over-expressed protein, there may be neuronal-specific or expression level-related effects that might account for the differences. For example, *Wld^S ^*has shown protection in several central nervous system models, but few have been further tested with only Nmnat1 even in dissociated neuronal models [37]. Then too, dopaminergic axons may have intrinsic differences that contribute to the *Wld^S ^*effect. For instance, dopamine neurons have fewer [68], smaller and slower mitochondria than non-dopaminergic neurons [19]. Moreover, dopamine neurons produce a neurotransmitter prone to oxidation [69], exhibit a greater dependence on L-type Ca^2+ ^channels with subunits that result in deleterious amounts of intracellular calcium and ensuing mitochondrial dysfunction [70], and extend long, thin lightly-myelinated processes which are selectively vulnerable in PD [71]. This suggests that dopaminergic neurons may be more vulnerable to insults that affect mitochondrial function. Given that enzymatically inactive *Wld^S ^*is able to protect dopaminergic, but not DRG neurons, it is possible that *Wld^S ^*also protects mitochondria in a manner independent of its NAD^+^-synthesizing ability. This as yet unknown function of *Wld^S ^*may be unmasked in dopaminergic neurons due to their unique phenotype. In contrast, NAD^+ ^may contribute more to *Wld^S^*-mediated protection in non-DA neurons.

## Conclusions

In support of our previous *in vivo *study showing that *Wld^S ^*protects dopaminergic terminal fields from MPTP, the current results demonstrate in dissociated dopamine cultures that the entire *Wld^S ^*sequence is needed for axonal protection, regardless of its NAD^+^-synthesizing activity. Indeed, NAD^+ ^and *Wld^S ^*act through separate, possibly parallel, mechanisms to protect dopamine axons. As MPP^+ ^is thought to impair mitochondrial function, in agreement with other studies, our results suggest that *Wld^S ^*might be involved in preserving mitochondrial health or maintaining cellular metabolism. Given that Parkinson's disease is the second most common neurodegenerative disorder, our findings support the idea that studies expanding therapeutic efforts towards maintaining connections as well as saving the cell body will help in developing better interventions for PD.

## Materials and methods

### Cell culture and toxin treatment

For primary midbrain cultures, the ventral mesencephalon was removed from embryonic day 14 (E14) murine embryos as previously described [33,72]. Wild-type (C57/Bl6) and *Wld^S ^*(C57Bl/OlaHsd-WldS) mice were ordered from Harlan (Bichester, UK). Sirt1 knockout mice were obtained from Dr. Christian Sheline (Louisiana State University - Health Science Center, New Orleans, LA). *Cyto Wld^S ^*mice were obtained from Dr. Michael Coleman (Babraham Institute, UK) [73]. Animals were treated in accordance with the National Institutes of Health *Guide for the Care and Use of Laboratory *Animals. All procedures were approved by the Washington University School of Medicine animal experimentation committee. Plates were pre-coated overnight with 0.2 mg/ml poly-D-lysine (Sigma-Aldrich, St. Louis, MO). Cells were plated at a density of approximately 125,000 cells/cm^2 ^and maintained in serum-free Neurobasal medium (Invitrogen, Carlsbad, CA) supplemented with 1× B27 supplement (Invitrogen), 0.5 mM L-glutamine (Sigma-Aldrich), and 0.01 μg/ml streptomycin plus 100 U penicillin. Half of the culture medium was replaced with fresh Neurobasal medium after 5 days *in vitro *(DIV). Cultures were pretreated with 1 mM NAD^+^, 1 mM NMN, 1 mM nicotinic acid mononucleotide (NaMN), or a comparable volume of vehicle 24 hours before toxin treatment. Cultures were treated with either 1 μM 1-methyl-4-phenylpyridinium (MPP+), the active metabolite of MPTP or vehicle on DIV 7. Dorsal root ganglion (DRG) cells were obtained from E14 murine embryos as previously described [74]. Cells were plated on coverslips precoated with 0.1 mg/ml poly-L-ornithine (Invitrogen) and 32 μg/ml laminin-1 (Invitrogen) and maintained in DRG media which consisted of Eagle Minimal Essential Media (Invitrogen) supplemented with chick embryo extract (Invitrogen), 10% fetal calf serum (Invitrogen), 50 ng/ml Nerve Growth Factor (Harlan Biosciences, Madison, WI) and 50 U/ml penicillin-50 g/ml streptomycin. Half of the culture medium was replaced with fresh DRG medium after DIV 5. After transduction with lentivirus on DIV 2, DRG cultures were treated with 0.4 μM vincristine or vehicle on DIV 7. NAD^+^, NMN, NaMN, MPP^+^, and vincristine were all obtained from Sigma-Aldrich.

### Lentiviral infection of dopaminergic neurons

The lentiviral expression plasmids FUGW, FCIV-Wld^S^, FCIV-Nmnat1, FCIV-Ube4b, FCIV-Nmnat3, FCIV- Nmnat1(W170A), FCIV-cytNmnat1, and FCIV-Wld^S^(W258A) were obtained from Dr. Jeffrey Milbrandt (Washington University, Saint Louis). Lentiviruses expressing transgenes were generated by the Hope Center for Neurological Disorders Viral Core (Washington University, Saint Louis). For infection of DRG and primary midbrain neurons, 50 μl lentivirus (10^5 ^infectious units/μl) was added to the well of a 7-mm dish containing approximately 70,000 neurons on DIV 2. Transduced primary midbrain and DRG neurons were treated with MPP^+ ^and vincristine, respectively, on DIV 7. Viral infection and transgene expression was monitored using the GFP reporter via fluorescent microscopy.

### Immunocytochemistry

Primary dopaminergic cultures and DRGs were plated in 7 mm microwell plates (MatTek Corp., Ashland, MA). Cells treated with MPP^+ ^were fixed with 4% paraformaldehyde (PFA) in PBS after 48 hours. Cultures were stained with sheep polyclonal anti-tyrosine hydroxylase (TH) (Novus Biologicals, Littleton, CO) and Cy3 α-sheep (Molecular Probes, Carlsbad, CA). Localization of cytoplasmic *Wld^S ^*was confirmed using rabbit *Wld^S ^*antibody (gift of M.P. Coleman) and Alexa488 α-rabbit (Molecular Probes). TH^+ ^cells and neurites were counted using unbiased stereological methods (Stereo Investigator, MicroBrightField, Williston, VT). DRG cultures treated with vincristine were subsequently stained with mouse acetylated tubulin (Sigma-Aldrich) and Cy3 α-mouse (Molecular Probes). Neurites were counted as described above. All images were acquired by confocal microscopy (Olympus Fluoview 500, Olympus, Center Valley, PA) and processed in ImageJ (NIH).

### Western Blotting

Primary midbrain cultures were plated in 48-well plates and transduced with the transgene of interest as described above. Lysates were collected in RIPA buffer (150 mM NaCl, 1% Nonidet P-40, 0.5% NaDoc, 0.1% SDS, 50 mM Tris pH 8.0) with protease inhibitor mixture (Roche, Mannheim, Germany) and incubated on ice for 30 minutes. Insoluble cell debris was removed by centrifugation and the protein concentration of each cell lysate was determined by Bradford protein assay (BioRad, Hercules, CA). Equal amounts of protein were run on SDS-polyacrylamide gels and transferred to polyvinylidene diflouride (PVDF) membranes (BioRad). PVDF membranes were probed with either rabbit *Wld^S ^*antibody or chicken polyclonal anti-GFP antibody (Aves Labs, Tigard, OR). As a control, PVDF membranes were also probed with goat polyclonal anti-HRP60 antibody (Santa Cruz Biotechnology, Santa Cruz, CA). The secondary antibodies used were either a HRP-linked rabbit antibody or HRP-linked anti-chicken antibody and a HRP-linked anti-goat antibody (Jackson Immunoresearch, West Grove, PA). Membranes were developed with enhanced chemiluminescence (Amersham Biosciences), imaged with either a Storm PhosphorImager (Molecular Dynamics) or a ChemiDoc XRS System (Bio-Rad, Hercules, CA) and band intensities were determined using ImageQuant software (Amersham Biosciences).

### Quantification of Cells and Neurites

TH^+ ^cells and neurites were counted using unbiased stereological methods [75] (Stereo Investigator (MicroBrightfield, Williston, VT), in combination with a Zeiss Axioplan2 microscope (Thornwood, NY) and an Optronics Microfire camera. The number of counting sites necessary to achieve a coefficient of error < 0.1 was determined by preliminary experiments. The total number of TH^+ ^cell bodies was calculated using the Fractionator function on Stereo Investigator by dividing the estimated number of cells by the estimated volume (μm^3^) of the dish sampled. Using the Petrimetrics function on Stereo Investigator, TH^+ ^neurites intersecting the boundary of the Petrimetric probe were counted. Neurite length was derived by dividing the total estimated neurite length (μm) by the estimated volume (μm^3^) of the dish sampled

### Statistical analysis

GraphPad Prism software (San Diego, CA) was used for statistical analysis. All data was collected from a minimum of three independent experiments done in triplicate. The significance of effects between control and experimental conditions was determined by a Student t-test or one-way ANOVA with Bonferroni Multiple Comparisons tests.

## Competing interests

The authors declare that they have no competing interests.

## Authors' contributions

JAD participated in experimental design, carried out all the experiments described and drafted the manuscript. KOM was involved in the design of experiments and production of the manuscript. Both authors participated in revising and editing the final manuscript. The final manuscript was read and approved by both authors.

## Supplementary Material

Additional file 1 **Figure S1 **- **Transduction efficiency of *Wld^S^*, Nmnat1 and Ube4b lentiviruses**. (A) Diagram of constructs used to transduce WT dissociated dopaminergic neurons. (B) Western blot of cell lysates from transduced primary midbrain cultures using the quantitative chemidoc imaging system with MAP2 as a loading control. Transduced constructs exhibited similar levels of expression. (C) Diagram of Nmnat1, inactive Nmnat1, cyto Nmnat1 and Nmnat3 lentiviral constructs used to transduce WT dissociated dopaminergic neurons. (D) Quantification of the western blots illustrates that these transgenes exhibit similar levels of expression. (E) Quantification of the western blots from the primary midbrain culture lysates of either WT mice, native *Wld^S ^*mice, of WT mice transduced with *Wld^S ^*virus. (E) Quantification of the western blots of brain lysates taken from either the substantia nigra (SN) or striatum (STR) of WT, native *Wld^S ^*mice, or Cyto *Wld^S ^*mice. Click here for file
