# Supplementary figures and images for: WldS but not Nmnat1 protects dopaminergic neurites from MPP+ neurotoxicity
Source: Mol Neurodegener. 2012 Feb 8;7:5. doi: 10.1186/1750-1326-7-5 (PMC3322348; doi:10.1186/1750-1326-7-5)

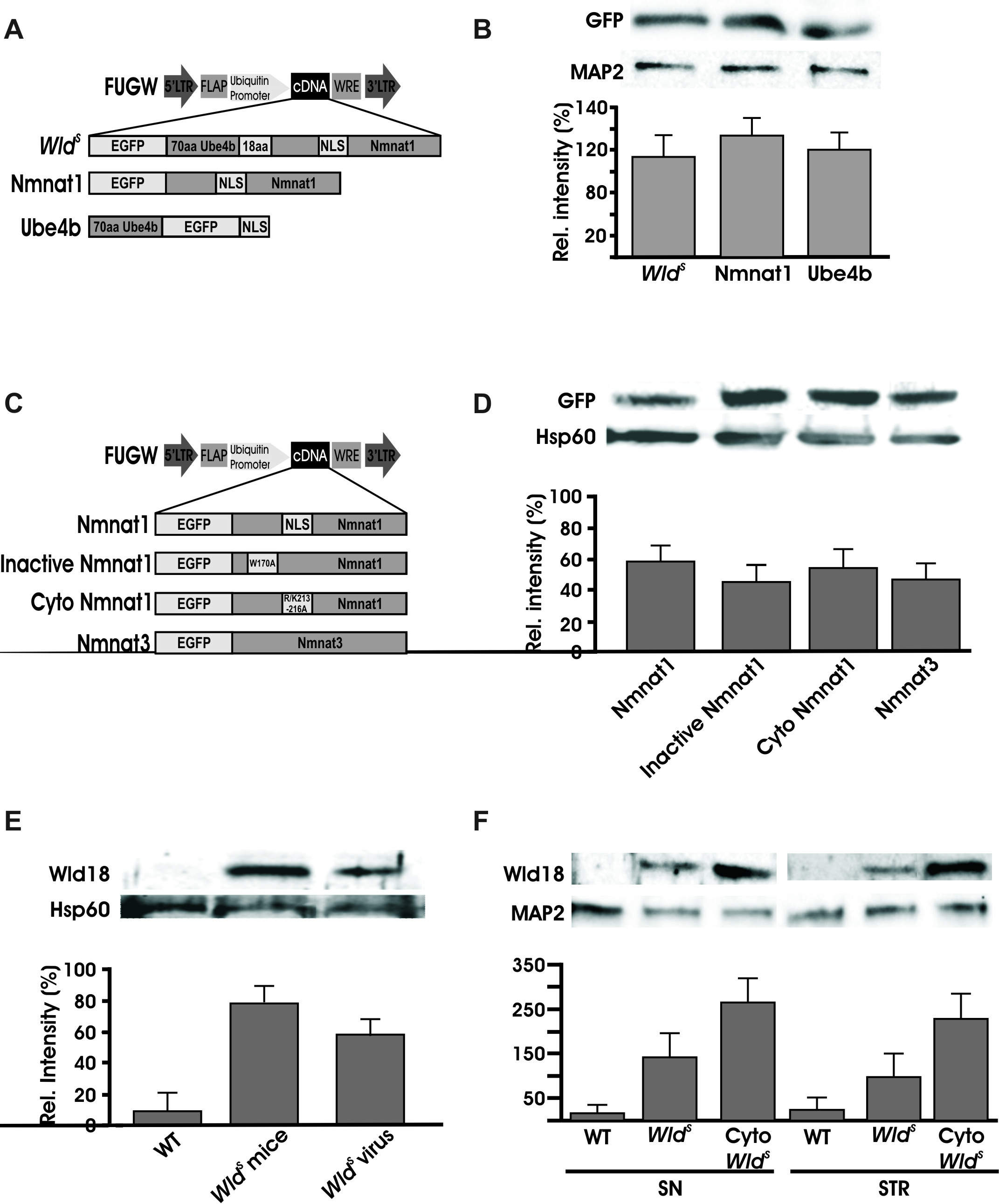

Supplement: Additional file 1 — Figure S1 - Transduction efficiency of WldS, Nmnat1 and Ube4b lentiviruses. (A) Diagram of constructs used to transduce WT dissociated dopaminergic neurons. (B) Western blot of cell lysates from transduced primary midbrain cultures using the quantitative chemidoc imaging system with MAP2 as a loading control. Transduced constructs exhibited similar levels of expression. (C) Diagram of Nmnat1, inactive Nmnat1, cyto Nmnat1 and Nmnat3 lentiviral constructs used to transduce WT dissociated dopaminergic neurons. (D) Quantification of the western blots illustrates that these transgenes exhibit similar levels of expression. (E) Quantification of the western blots from the primary midbrain culture lysates of either WT mice, native WldS mice, of WT mice transduced with WldS virus. (E) Quantification of the western blots of brain lysates taken from either the substantia nigra (SN) or striatum (STR) of WT, native WldS mice, or Cyto WldS mice. [file 1750-1326-7-5-S1.JPEG]
